# Transcriptome analysis of the hippocampus in environmental noise-exposed SAMP8 mice reveals regulatory pathways associated with Alzheimer’s disease neuropathology

**DOI:** 10.1186/s12199-019-0840-6

**Published:** 2020-01-09

**Authors:** Donghong Su, Wenlong Li, Huimin Chi, Honglian Yang, Xiaojun She, Kun Wang, Xiujie Gao, Kefeng Ma, Ming Zhang, Bo Cui

**Affiliations:** 1 Tianjin Institute of Environmental and Operational Medicine, Tianjin, China; 2 School of Public Health and Management, Weifang Medical University, Weifang, China; 3 Tianjin Centers for Disease Control and Prevention, Tianjin, China

**Keywords:** Environmental noise, Alzheimer’s disease, RNA sequencing, SAMP8 mice

## Abstract

**Background:**

Chronic noise exposure is one environmental hazard that is associated with genetic susceptibility factors that increase Alzheimer’s disease (AD) pathogenesis. However, the comprehensive understanding of the link between chronic noise stress and AD is limited. Herein, we investigated the effects of chronic noise exposure on AD-like changes in senescence-accelerated mouse prone 8 (SAMP8).

**Methods:**

A total of 30 male SAMP8 mice were randomly divided into the noise-exposed group, the control group, and aging group (positive controls), and mice in the exposure group were exposed to 98 dB SPL white noise for 30 consecutive days. Transcriptome analysis and AD-like neuropathology of hippocampus were examined by RNA sequencing and immunoblotting. Enzyme-linked immunosorbent assay and real-time PCR were used to further determine the differential gene expression and explore the underlying mechanisms of chronic noise exposure in relation to AD at the genome level.

**Results:**

Chronic noise exposure led to amyloid beta accumulation and increased the hyperphosphorylation of tau at the Ser202 and Ser404 sites in young SAMP8 mice; similar observations were noted in aging SAMP8 mice. We identified 21 protein-coding transcripts that were differentially expressed: 6 were downregulated and 15 were upregulated after chronic noise exposure; 8 genes were related to AD. qPCR results indicated that the expression of Arc, Egr1, Egr2, Fos, Nauk1, and Per2 were significantly high in the noise exposure group. These outcomes mirrored the results of the RNA sequencing data.

**Conclusions:**

These findings further revealed that chronic noise exposure exacerbated aging-like impairment in the hippocampus of the SAMP8 mice and that the protein-coding transcripts discovered in the study may be key candidate regulators involved in environment-gene interactions.

## Introduction

Alzheimer’s disease (AD) is the most common form of dementia, accounting for 50–56% of autopsy and clinical cases [1]. It is marked by a progressive loss of memory and cognitive function, overproduction of amyloid beta (Aβ), and increased hyperphosphorylation of tau [1, 2]. However, the pathogenesis of AD is complex, and the disease has no clear cause.

Generally, the association among environmental stress, aging process, and their causal roles plays a role in the development of AD pathogenesis [3–5]. Aging is the major risk factor for AD [6]. The incidence of dementia increases exponentially with increasing age [7]. AD-like pathological changes, such as aggregation of Aβ and the phosphorylation of tau protein, are associated with aging [8, 9]. Noise-induced health effects were first recognized in industrial settings, such as manufacturers, where intense impulse sound or steady-state long-term exposure with sound pressure levels higher than 85 dBA were associated with auditory and non-auditory effects [10]. Chronic noise exposure in experimental animals can cause not only accelerated Aβ synthesis but also significant and persistent hyperphosphorylation of tau and the formation of prominent pathological neurofibrillary tangles (NFTs) of tau in the hippocampus, key structures in learning and memory, and initial sites of tau pathology in AD [11–14]. On the other hand, chronic noise exposure could affect the spatial learning and memory [15, 16]. Hence, noise exposure may aggravate the development of aging-related AD-like pathological changes, and its related molecular mechanisms need to be explored further.

In the present study, we performed a comparative gene expression analysis of chronic noise-affected and non-noise-affected brain tissues by using the RNA sequencing technique, an advanced approach that determines the differential expression profiles underlying phenotypic differences [17]. We selected senescence-accelerated mouse prone 8 (SAMP8), which shares behavioral, cognitive, and neuropathological alterations observed in AD patients and is a plausible natural model for exploring the pathogenesis of AD [18]. Our data will serve as a new reference for the prevention and treatment of AD.

## Materials and methods

### Animal use and experimental grouping

The 30 male SAMP8 mice used in our study were provided by the Tianjin University of Traditional Chinese Medicine. The mice were kept under standard housing conditions with controlled ambient temperature (23 ± 2 °C) and humidity (50–60%) and a 12-h light/12-h dark cycle (lights on from 06:00 to 18:00). Food and water were provided in their home cages, and they were allowed to adapt to the laboratory environment for 5 days before carrying out the experiment.

Three-month-old male SAMP8 mice were randomly separated into the control group (control group) and noise exposure group exposed to 98 dB SPL white noise (noise group). Eight-month-old male SAMP8 mice were used as positive controls (aging group). The number of animals in each group is equal. The noise group was exposed to 98 dB SPL white noise from 8:00 to 12:00 for 30 days while the control group and the aging group housed in similar cages were exposed to background noise (< 40 dB SPL) from another chamber. After 30 consecutive days of exposure to noise, the mice were sacrificed, and brain samples were immediately collected for biochemical analyses and stored at − 80 °C until use. All experiments adhered to the guidelines of the National Institute of Health for the use of experimental animals and were performed in accordance with the approved guidelines specified by the Animal and Human Use in Research Committee of the Tianjin Institute of Environmental Medicine and Operational Medicine.

### Noise exposure setup

All noise exposures were performed as previously described [19]. Noise was generated using a noise generator (BK 3560C, B&K Instruments, Denmark), amplified using a power amplifier (Yong-Sheng Audio P-150D; The Third Institute of China Electronics Technology Group, Beijing, China), and transmitted through a loudspeaker (ZM-16S; Tianjin Zenmay Electroacoustic Equipment Co., Tianjin, China). The frequency of the noise emitted from the speaker was in the range of 200–6300 Hz (1/3 octave bands). Animals were exposed to noise in a reverberation chamber, where wire-mesh cages were placed at the center of the sound field. A loudspeaker was suspended directly above the cages. The noise level variation was less than 2 dB in the available space of the animals. The background noise level in the chamber was below 40 dB SPL.

### Detection of Aβ by enzyme-linked immunosorbent assay

Frozen mouse hippocampus was homogenized in ice-cold 1× phosphate-buffered saline (0.02 mol/L, pH 7.0–7.2). Aβ1-40 and Aβ1-42 concentrations were measured in soluble and insoluble brain fractions with mouse ELISA kits (BlueGene Biotech, Shanghai, China) in accordance with the manufacturer’s instructions. The mean value of the duplicate samples was considered as the final concentration for each animal.

### Western blot analysis

Hippocampus preparation and Western blot were performed as described previously [13, 20]. Briefly, each hippocampus was dissected immediately after sacrificing the animal and was stored at − 80 °C until use. For immunoblot analysis, the frozen hippocampi were homogenized in ice-cold 50 mM Tris-HCl buffer (pH 7.4) containing 1% Triton X-100, 0.2 mM PMSF, and 1 mM EDTA. Homogenates were centrifuged at 12,000×*g* for 10 min at 4 °C. The supernatants obtained were immediately placed in boiling water for 10 min. Samples (10 μg protein/lane) were separated on 10% SDS-PAGE gels and electrophoretically transferred to nitrocellulose membranes. The membranes were probed with rabbit antibodies against the following proteins: Tau (C-17) (polyclonal, 1:1000, Bioworld Technology, China), PS202 (polyclonal, 1:1000, Bioworld Technology, China), PS404 (polyclonal, 1:1000, Bioworld Technology, China), Egr1 (polyclonal, 1:1000, Bioworld Technology, China), and c-Fos (polyclonal, 1:1000, Bioworld Technology, China). Anti-GAPDH (1:10,000; Bioworld Technology) was used as the internal reference standard.

### RNA quantification and qualification

RNA degradation and contamination were monitored using 1% agarose gel. RNA purity was checked using the NanoPhotometer® spectrophotometer (IMPLEN, CA, USA). RNA concentration was measured using the Qubit® RNA assay kit in Qubit® 2.0 Fluorometer (Life Technologies, CA, USA). RNA integrity was assessed using the RNA Nano 6000 assay kit of the Bioanalyzer 2100 system (Agilent Technologies, CA, USA).

### Library preparation for transcriptome sequencing

For the RNA sample preparations, 3 μg RNA per sample was used as input material. Sequencing libraries were generated using NEBNext® UltraTM RNA library prep kit for Illumina® (NEB, USA), and index codes were added to attribute sequences to each sample. Briefly, mRNA was purified from total RNA by using poly-T oligo-attached magnetic beads. Fragmentation was carried out using divalent cations under elevated temperature in NEBNext first-strand synthesis reaction buffer (5×). Clustering was performed on a cBot cluster generation system by using TruSeq PE cluster kit v3-cBot-HS (Illumina). The RNA integrity number (RIN) of all samples was greater than 6.8.

### Read mapping to the reference genome

Reference genome and gene model annotation files were directly downloaded from the genome website. The index of the reference genome was built using Hisat2 v2.0.5, and paired-end clean reads were aligned to the reference genome by using Hisat2 v2.0.5 (ftp://ftp.ensembl.org/pub/release-94/gtf/mus_musculus/). We selected Hisat2 as the mapping tool to allow Hisat2 to generate a database of splice junctions on the basis of the gene model annotation file and obtain better mapping results than those obtained by other non-splice mapping tools.

### Quantification of gene expression level

FeatureCounts v1.5.0-p3 was used to count the read numbers mapped to each gene. Then, the fragments per kilobase of transcript sequence per million (FPKM) of each gene were calculated on the basis of the length of the gene and the number of reads mapped to this gene. The method of using the expected number of FPKM base pairs sequenced considers the effects of sequencing depth and gene length on the read count and is currently the most commonly used technique for estimating gene expression levels. The correlations between samples were evaluated using Pearson’s correlation coefficient (Additional file 1: Figure S1, A). And the principal component analysis (PCA) was used to assess the inter-group differences and the repetition of the samples within the group (Additional file 1: Figure S1, B).

### Differential expression analysis

Differential expression analysis of two conditions/groups (two biological replicates per condition) was performed using the DESeq2 R package (1.16.1). DESeq2 provides statistical routines for determining the differential expression in the digital gene expression data by using a model based on a negative binomial distribution. The resulting *P* values were adjusted using the Benjamini and Hochberg’s approach for controlling false discovery rates. Genes with an adjusted *P* value < 0.05 found by DESeq2 were regarded as differentially expressed.

### Gene Ontology and Kyoto Encyclopedia of Genes and Genomes enrichment analysis of differentially expressed genes

GO enrichment analysis of differentially expressed genes was implemented using the cluster Profiler R package, in which gene length bias was corrected. GO terms with corrected *P* value < 0.05 were considered significantly enriched by differentially expressed genes. KEGG is a database resource for understanding the high-level functions and utilities of the biological system. We used the cluster Profiler R package to test the statistical enrichment of the differentially expressed genes in the KEGG pathways. Environmental noise-related genes and pathways were analyzed by Gene Ontology (GO) and Pathway-Relation-Network (Path-net) analysis tools based on Kyoto Encyclopedia of Genes and Genomes (KEGG) Pathway Database using Gene Cloud of Biotechnology Information (GCBI Platform, Shanghai, China) (www.gcbi.com.cn).

### Determination of gene expression by real-time PCR

Real-time PCR was performed as previously described [20]. The hippocampus tissues from the exposure and control group mice were homogenized using a rapidly oscillating masher. Total RNA was extracted using an RNeasy Mini kit (TaKaRa Bio, Dalian, China) according to the manufacturer’s protocol. Total RNA was converted to cDNA via reverse transcription by using a transcriptor first-strand cDNA synthesis kit (TaKaRa Bio, Dalian, China). A primer pair designed to amplify the *GAPDH* gene was used as an internal control. Specific primers and probes designed for mouse *Arc*, *Egr1*, *Egr2*, *c-Fos*, *Hmgcr*, *Hap1*, *Nauk1*, *Per2*, and *GAPDH* were used as described in Table 1. Gene expression levels were assessed by quantitative real-time PCR under the following thermal cycling conditions: 2 min at 50 °C and 10 min at 95 °C followed by 45 cycles of 95 °C for 5 s and 57 °C for 30 s. Real-time PCR was performed using gene expression assays-on-demand and a Takara PCR thermal cycler dice real-time system (TaKaRa Bio, Dalian, China). The threshold cycle (Ct) of the target genes was normalized to that of GAPDH. mRNA levels in noise-exposed animals were calculated after normalizing the cycle thresholds to the GAPDH expression and were presented as fold-induction values (2^−ΔΔCt^) relative to the control mice.

**Table 1:** Mouse primers used for quantitative real-time PCR

Gene	Primers
Egr2	F:5'-GGAGAGAGTCAGTGACGGATAGA-3' R:5'-TTTGCTCCTCGCACAACCTG-3'
Egr1	F:5'-CCACCATGGACAACTACCCC-3' R:5'-TCATAGGGTTGTTCGCTCGG-3'
Fos	F:5'-TACTACCATTCCCCAGCCGA-3' R:5'-GCTGTCACCGTGGGGATAAA-3'
Hap1	F:5'-GCCCATCTAGAAACCCCAGC-3' R:5'-AGGGCCATGAAGACGAAAGG-3'
Nauk1	F:5'-GGACGAGCTAGACATGGTTCA-3' R:5'-AGTAATGCACGGCAGACACA-3
Per2	F:5'-CCACTATGTGACAGCGGAGG-3' R:5'-TGTCGGGCTCTGGAATAAGC-3'
Hmgcr	F:5'-TGAGATCCGGAGGATCCAAGG-3' R:5'-CAGATCTTGTTGTTGCCGGTG-3'
Arc	F:5'-TGGAGGGAGGTCTTCTACCG-3' R:5'-CCTACAGAGACAGTGTGGCG-3
GAPDH	F:5'-AGGTCGGTGTGAACGGATTTG-3' R:5'-TGTAGACCATGTAGTTGAGGTCA-3'

### Statistics

Results were expressed as mean ± standard deviation of the mean. Data were analyzed using the SPSS v.19.0 software (SPSS Inc., Chicago, IL, USA). Data were subjected to one-way analysis of variance, followed by the least significant difference method test or Dunnett’s *t* test. Values were considered to be significantly different at *P* < 0.05.

## Results

### Chronic noise exacerbates AD-like neuropathology in SAMP8 mice

**Fig. 1:**
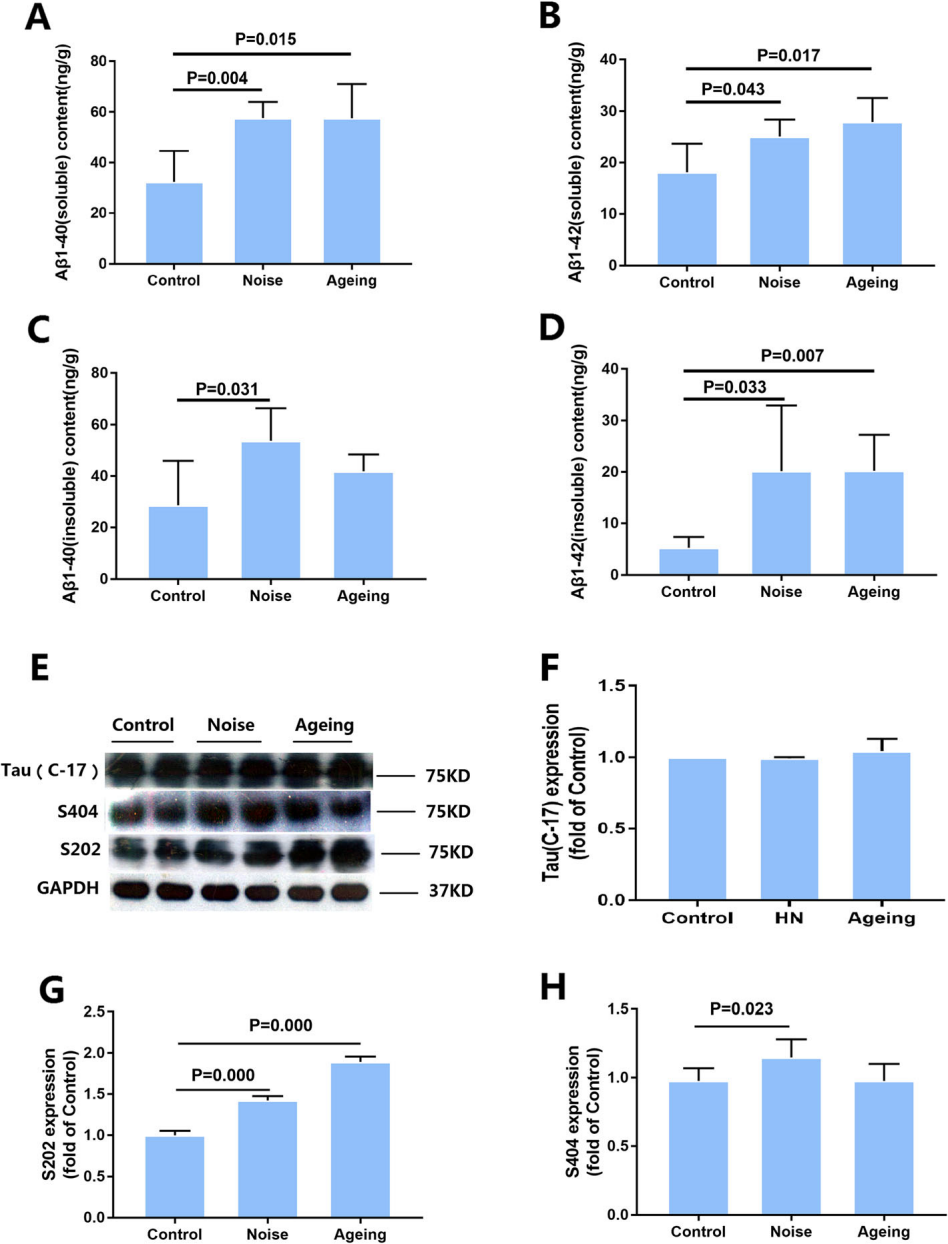
Chronic noise-induced Alzheimer’s disease (AD)-like pathological alterations in SAMP8 mice. **a**–**d** Quantification of soluble and insoluble Aβ1-40 and Aβ1-42 levels by ELISA. **e** The expression of total tau and hyperphosphorylated tau in SAMP8 mice. **f**–**h** The density of the immunoreactive bands was quantified and represented as a percent change relative to the control. GAPDH was used as a loading control. Data were expressed as the mean ± standard deviation (*n* = 6 per group). Results were normalized as the control = 100%

To explore the effects of chronic noise exposure on Aβ in the hippocampus, we determined the concentrations of soluble and insoluble Aβ1-40 and Aβ1-42 in the brain components by using a mouse ELISA kit. The results showed that the amount of Aβ1-40 or Aβ1-42 in the soluble or insoluble fractions was significantly increased in the noise group relative to the control group and was close to that of the aging group (Fig. 1a–d).

Tau phosphorylation levels were assessed by quantitative immunoblot analyses of cortical extracts from individual mouse in each group after the end of the 30-day experiment period (Fig. 1e, f). In comparison with those in the control group, the levels of tau phosphorylated at Ser202 and Ser404 in the noise group were significantly increased (Fig. 1g, h). These data suggested that chronic noise exposure accelerated AD-like pathological alterations in the SAMP8 mice.

### Functional enrichment analysis

A total of 161,813,829 raw reads (80,365,338 for the noise group and 81,448,491 for the control group) were generated. After discarding the reads with adapters, poly-N > 10%, and any other possible contaminants, 156,995,998 clean reads (78,392,394 for the noise group and 78,603,604 for the control group) were obtained. The clean reads were mapped to the mouse reference genome, and the mapping rates were approximately 92.93% and 93.53% for the noise and control group mice, respectively. The cufflink results indicated 30,673 protein-coding transcripts. These mRNAs were used for subsequent analysis.

To identify significant associations of genes with any specific molecular pathway, we performed annotation enrichment analyses. Genes that significantly enriched for *P* value in GO terms included transmembrane receptor protein serine/threonine kinase signaling pathway, synaptic transmission (GABAergic), sleep and regulation of synaptic transmission (GABAergic), and several terms were closely related to AD, such as learning or memory (GO: 0007611 ), cognition (GO: 0050890), learning (GO: 0007612), and regulation of neuronal synaptic plasticity (GO: 0048168) (Table 2). By contrast, the KEGG results revealed 20 pathways, of which had significant *P* values included Toll-like receptor signaling pathway, TNF signaling pathway, and MAPK signaling pathway (Glutamatergic synapse) (Table 3). We also observed that Egr1, Fos, Egr2, and Arc were the main central genes, and they directly interacted with each other in the gene network analysis.

**Table 2:** Chronic noise exposure Significantly affected gene ontology

Go ID	Go Description	P value	Overlap gene name
GO:0007611	learning or memory	0.000154	Egr2; Fos;Hmgcr;Arc
GO:0050890	cognition	0.000235	Egr2; Fos; Hmgcr; Arc
GO:0007612	learning	0.000598	Fos; Hmgcr; Arc
GO:0048168	regulation of neuronal synaptic plasticity	0.001688	Egr2; Arc
GO:0007215	glutamate receptor signaling pathway	0.003899	Homer1; Arc
GO:0008306	associative learning	0.004507	Fos; Hmgcr
GO:0007568	aging	0.004563	Fos; Nuak1; Hmgcr
GO:0050804	modulation of synaptic transmission	0.004607	Egr2; Hap1; Arc
GO:0045073	regulation of chemokine biosynthetic process	0.011953	Egr1
GO:1902992	negative regulation of amyloid precursor protein catabolic process	0.011953	Hap1
GO:0048167	regulation of synaptic plasticity	0.014307	Egr2; Arc
GO:0032230	positive regulation of synaptic transmission, GABAergic	0.015513	Hap1
GO:0007216	G-protein coupled glutamate receptor signaling pathway	0.017879	Homer1
GO:1902003	regulation of beta-amyloid formation	0.017879	Hap1
GO:1902991	regulation of amyloid precursor protein catabolic process	0.021418	Hap1
GO:0034205	beta-amyloid formation	0.022595	Hap1
GO:2000311	regulation of AMPA receptor activity	0.024945	Arc
GO:0032731	positive regulation of interleukin-1 beta production	0.030797	Egr1
GO:1900271	regulation of long-term synaptic potentiation	0.031963	Arc
GO:0050435	beta-amyloid metabolic process	0.033128	Hap1
GO:0032732	positive regulation of interleukin-1 production	0.034292	Egr1
GO:2000772	regulation of cellular senescence	0.034292	Nuak1
GO:0030431	sleep	0.035454	Fos
GO:0032228	regulation of synaptic transmission, GABAergic	0.038933	Hap1
GO:0043666	regulation of phosphoprotein phosphatase activity	0.0424	Nuak1
GO:0090342	regulation of cell aging	0.0424	Nuak1
GO:1900449	regulation of glutamate receptor signaling pathway	0.0424	Arc
GO:1901214	regulation of neuron death	0.04305	Fos; Egr1
GO:0007178	transmembrane receptor protein serine/threonine kinase signaling pathway	0.044463	Fos; Egr1
GO:0042982	amyloid precursor protein metabolic process	0.044705	Hap1
GO:0099601	regulation of neurotransmitter receptor activity	0.044705	Arc
GO:0051932	synaptic transmission, GABAergic	0.047004	Hap1
GO:0032651	regulation of interleukin-1 beta production	0.048152	Egr1

**Table 3:** Chronic noise exposure significantly affected signaling pathways

Path ID	Path description	P value	Gene name	KEGG_ID
mmu04727	GABAergic synapse	0.170713	Hap1	mmu:15114
mmu04620	Toll-like receptor signaling pathway	0.172572	Fos	mmu:14281
mmu04933	AGE-RAGE signaling pathway in diabetic complications	0.199989	Egr1	mmu:13653
mmu04668	TNF signaling pathway	0.205371	Fos	mmu:14281
mmu04724	Glutamatergic synapse	0.217796	Homer1	mmu:26556
mmu04725	Cholinergic synapse	0.219557	Fos	mmu:14281
mmu04152	AMPK signaling pathway	0.231776	Hmgcr	mmu:15357
mmu04728	Dopaminergic synapse	0.245524	Fos	mmu:14281
mmu04024	cAMP signaling pathway	0.343077	Fos	mmu:14281
mmu04010	MAPK signaling pathway	0.476833	Fos	mmu:14281

### Differential expression analysis

**Fig. 2:**
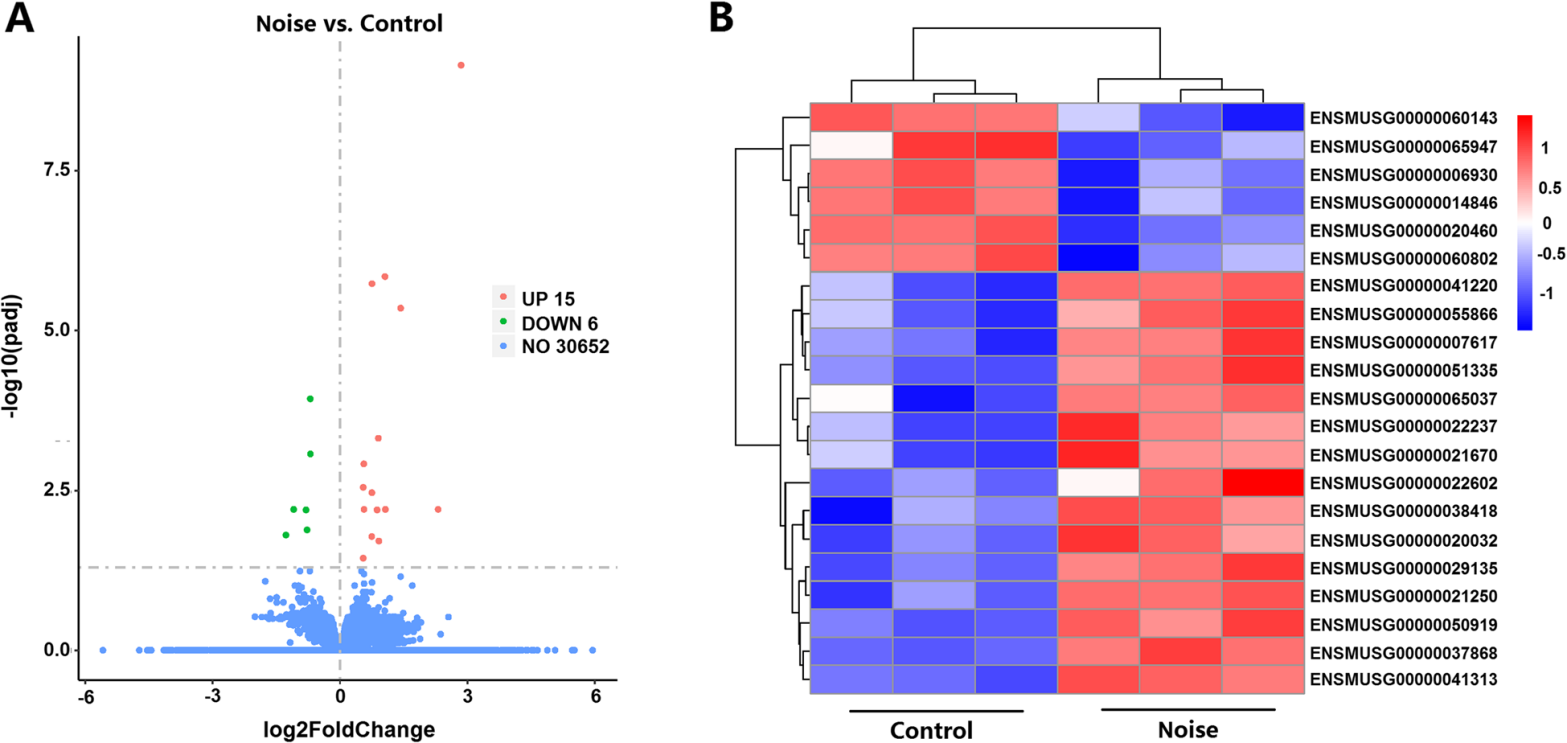
Cluster analysis shows the differentially expressed mRNAs. **a** Differentially expressed mRNAs by volcano map. The red markings indicate upregulated expression, and the green markings indicate downregulated expression. **b** Differentially expressed mRNAs by heat map. The red markings indicate an upregulated expression, while the blue markings indicate a downregulated expression

The mRNA expression levels and transcripts were estimated using FPKMs. A total of 30,673 mRNA transcripts were obtained. Expression analysis showed that 21 mRNA transcripts, including 15 upregulated and 6 downregulated transcripts (*P* < 0.05), were differentially expressed in the noise group relative to the control group (Table 4). Differentially expressed mRNAs were analyzed using the volcano map and clustering heat map (Fig. 2a, b). Exactly 8 of the 21 differentially expressed genes were related to AD (Table 5).

**Table 4:** Twenty-one significantly dysregulated mRNAs in the noise-exposed and control groups

Gene	Transcript ID	Foldchange	Description
Egr2	ENSMUSG00000037868	7.249386	early growth response 2
Homer1	ENSMUSG00000007617	2.090347	homer scaffolding protein 1
Fos12	ENSMUSG00000029135	1.690786	fos-like antigen 2
Fos	ENSMUSG00000021250	2.703889	FBJ osteosarcoma oncogene
Rps27a	ENSMUSG00000020460	0.617494	ribosomal protein S27A
Egr1	ENSMUSG00000038418	1.877242	early growth response 1
Hap1	ENSMUSG00000006930	0.619126	huntingtin-associated protein 1
Gfod1	ENSMUSG00000051335	1.48084	glucose-fructose oxidoreductase domain
Slc7a1	ENSMUSG00000041313	1.46453	solute carrier family 7
Ankrd33b	ENSMUSG00000022237	1.689168	ankyrin repeat domain 33B
Nuak1	ENSMUSG00000020032	1.485235	NUAK family, SNF1-like kinase,1
Gm10076	ENSMUSG00000060143	0.470904	predicted gene 10076
Rn7sk	ENSMUSG00000065037	4.981484	RNA, 7SK, nuclear
Zfp366	ENSMUSG00000050919	2.106077	zinc finger protein 366
B2m	ENSMUSG00000060802	0.575968	beta-2 microglobulin
Per2	ENSMUSG00000055866	1.838931	period circadian clock 2
Tppp3	ENSMUSG00000014846	0.585384	tubulin polymerization-promoting protein
mt-Nd41	ENSMUSG00000065947	0.414951	mitochondrially encoded NADH
Hmgcr	ENSMUSG00000021670	1.685124	3-hydroxy-3-methylglutaryl-Coenzyme A
Arc	ENSMUSG00000022602	1.892969	activity regulated cytoskeletal-associated protein
Elov16	ENSMUSG00000041220	1.465985	ELOVL family member 6, elongation of long chain fatty acids (yeast)

**Table 5:** AD-related genes identified after chronic noise exposure

Gene	Transcript ID	Foldchange
Egr2	ENSMUSG00000037868	7.25
Fos	ENSMUSG00000021250	2.70
Arc	ENSMUSG00000022602	1.89
Egr1	ENSMUSG00000038418	1.88
Per2	ENSMUSG00000055866	1.83
Hmgcr	ENSMUSG00000021670	1.69
Nauk1	ENSMUSG00000020032	1.49
Hap1	ENSMUSG00000006930	0.62

### Validation and quantitative assessment of selected AD-related differential genes

**Fig. 3:**
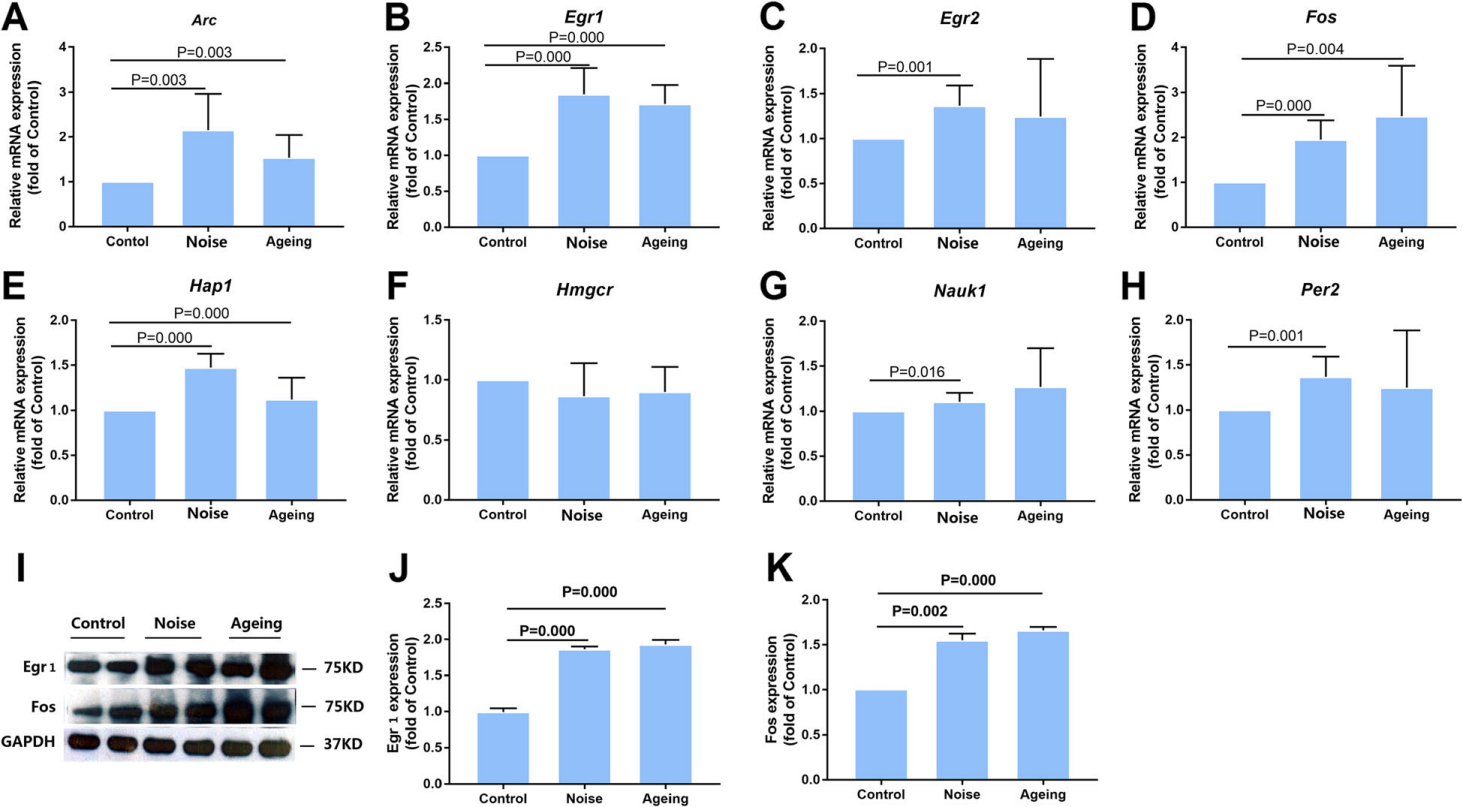
Validation and quantitative assessment of selected AD-related differential genes. **a**–**h**. Validation of transcript expression by qPCR. mRNA expression levels of differential genes in SAMP8 mouse brain samples; GAPDH was used as a loading control. **i**–**k**. Quantitative assessment of Egr1 and Fos levels in the hippocampus. The density of the immunoreactive bands was quantified and represented as a percent change relative to the control; GAPDH was used as a loading control. Data were expressed as mean ± standard deviation (*n* = 6 per group). Results were normalized with the control = 100%

Eight AD-related differentially expressed mRNA transcripts were selected to validate the accuracy of RNA sequencing via qPCR (Fig. 3a–h). The noise-exposed groups exhibited significantly higher mRNA expression of *Arc*, *Egr1*, *Egr2*, *Fos*, *Nauk1*, and *Per2* than the control mice, thereby confirming the results of the RNA sequencing data. The mRNA expression of *Arc*, *Egr1*, and *Fos* was also markedly higher in the aging mice than in the control mice; the mRNA expression in the latter was similar to that in the noise-exposed mice. No between-group differences were observed in the mRNA expression of *Hmgcr*. The mRNA expression of *Hap1* significantly increased in the noise and aging groups, whereas it decreased in the RNA sequencing. To further validate the accuracy of the RNA sequencing, the *Egr1* and *Fos* levels were assessed by Western blot analysis. Immunoblotting confirmed that the expressions of Egr1 and Fos increased more in the noise-exposed and aging mice than in the control mice (Fig. 3i–k). These results indicated that chronic noise exposure induced aging-like impairment in the hippocampi of the SAMP8 mice.

## Discussion

Multiple factors contribute to the pathogenesis of AD, and they include aging, genetic variables, and environmental factors [21]. Chronic noise exposure, the main focus of environmental factors, is related to AD [12–14, 22]. Acute or chronic noise exposure could induce various indicators of AD-like pathological changes that increase the risk of AD development [19, 23]. Our recent research further confirmed that chronic noise exposure led to cognitive impairment and Aβ accumulation in young SAMP8 mice, similar to that observed in aging SAMP8 mice [12]. In the present study, we described a reliable transcriptome sequencing to highlight key proteins in the hippocampus that are modulated in response to AD-like neurodegenerations. We also reported genomic signatures that are associated with chronic noise exposure and with age-related changes in the tissue.

By overlaying transcriptomic and neuropathological profiles, we identified noise and age-related shifts in underappreciated pathways, such as the Toll-like receptor (TLR) signaling pathway, TNF signaling pathway, MAP kinase pathway, and Ras/ERK signaling pathway, which are involved in the pathogenesis of AD. TLRs, a family of receptor proteins, play a wide role in innate and adaptive immune responses upon the stimulations by exogenous and endogenous TLR ligands. There is an increased endocytosis of TLR4 in the AD model mice brain, which might be a key event for the neurodegeneration signaling in the brain [24]. TNF-α has been assessed in the pathophysiology of AD both in human [25] and animal studies [26], and in our previous study, chronic noise exposure increased levels of TNF-α in the rat hippocampus [20]. The mammalian MAPK family consists of p38-MAPK, ERK, and c-Jun NH2-terminal kinase (JNK), which is involved in inflammatory reactions and apoptosis processes under conditions of oxidative stress [27]. It was also reported in our previous study that noise exposure could induce neural apoptosis [28], which is influenced by multiple factors and was shown to be closely related to the MAPK pathway [29]. AD is a multifactorial disease, and many theories have been formulated concerning its causes, including neuron loss, Aβ deposition, tau neuropathology, immune system dysfunction, synapse injury, oxidative stress, and mitochondrial dysfunction [30]. In AD, the differential expression of genes can affect different signaling pathways [31]. As expected, our analysis showed that chronic noise exerted a multi-level effect on the acceleration of AD progress, which indicated that chronic noise exposure accelerated AD progress to a certain extent.

Our finding that AD-related genes [32–36], including *Egr2*, *Fos*, *Arc*, *Hmgcr*, *Nuak1*, *Egr1*, *Hap1*, *Per2*. *Egr2*, Fos, *Arc*, and *Hmgcr*, show significant differential expression in noise-exposed mice lends support to the idea that environmental hazards including noise exposure may promote the occurrence and development of AD. Egr is a transcription factor and belongs to the family of immediate early genes induced by serum, which is closely related to the differentiation of some tissues and cells and is expressed in the early stage of central nervous system development, which promotes the formation of afterbrain. Upregulation of phagocytic markers Egr2 was observed in Aβ plaque-associated microglia, which reflects an attempt to enhance phagocytosis in plaque-associated microglia [37]. *Egr-1* mRNA level is fourfold higher in AD brain than in non-AD brain, and the *Egr-1* level is high in brain areas with high NFT density [38]. The overexpression of Egr-1 in rat brain could promote tau phosphorylation at Ser396/404 and Ser262 [39]. In the present study, we found an increase in the phosphorylation levels of tau protein at the Ser202 and Ser404 sites in the noise and aging groups. These alterations were also concomitant with the overexpression of Egr-1 after the end of the stimulus. Furthermore, lasting increase in Fos was found to occur in close correspondence with increase in tau hyperphosphorylation.

Aβ precursor protein (APP) metabolism engages neuronal endolysosomal pathways for Aβ processing and secretion. In AD, the dysregulation of APP leads to excess Aβ. Hap1 may promote the trafficking of APP into the non-amyloidogenic pathway and reduce the production of Aβ [40]. In the present study, the amount of Aβ increased when Hap1 was decreased; hence, regulating Hap1 expression may help to control Aβ production and affect the development of AD. In our previous study, following noise stress, we detected significantly higher levels not only in Aβ but also in the phosphorylation of tau [16, 17, 28, 41]. Our findings indicate that the long-lasting changes in tau phosphorylation observed after chronic noise exposure are likely to be the result of a complex regulatory network of multiple signaling pathways.

Due to the consideration of the influence of too many factors on the results of transcriptome, we did not use wild-type mice to set up the normal control group, which may affect the in-depth analysis of the research results. Additionally, the dose-response relationship of noise exposure was not considered in this study. More perfect experiments will be designed according to the important clues and limitations of the present study.

## Conclusion

In summary, our results show the exacerbation of aging-like impairment following chronic noise exposure, as well as candidate genes mediating AD-like pathological changes in the hippocampus of noise-exposed SAMP8 mice. We also identify significant associations of genes with specific molecular pathway, which may be key candidate regulators involved in environment-gene interactions, and suggest that a more detailed look into the underlying mechanism of AD pathogenesis is warranted. Further detailed studies are required to clarify the molecular mechanisms underlying the regulation of the multifaceted signaling system in AD etiology.

## Supplementary information

**Additional file 1: Figure S1.** *Sample overview of data characteristics. A* The correlations between samples. *B* The inter-group differences and the repetition of the samples within the group.

## Data Availability

All data generated or analyzed during this study are included in this published article.

## Abbreviations

AD: Alzheimer’s disease
APP: Aβ precursor protein
Aβ: Amyloid beta
ELISA: Enzyme-linked immunosorbent assay
FPKM: Fragments per kilobase of transcript sequence per million
GO: Gene ontology
KEGG: Kyoto Encyclopedia of Genes and Genomes
NFTs: Neurofibrillary tangles
PCA: Principal component analysis
SAMP8: Senescence-accelerated mouse prone 8
SPL: Sound pressure level
TBST: Tris-buffered saline-Tween
